# A national survey of health service infrastructure and policy impacts on access to computerised CBT in Scotland

**DOI:** 10.1186/1472-6947-12-102

**Published:** 2012-09-08

**Authors:** David Kenicer, Carrie-Anne McClay, Christopher Williams

**Affiliations:** 1 Gartnavel Royal Hospital, Glasgow, UK; 2 Academic Unit of Mental Health and Wellbeing, Institute of Health and Wellbeing, University of Glasgow, Gartnavel Royal Hospital, Glasgow, UK

## Abstract

**Background:**

NICE recommends computerised cognitive behavioural therapy (cCBT) for the treatment of several mental health problems such as anxiety and depression. cCBT may be one way that services can reduce waiting lists and improve capacity and efficiency. However, there is some doubt about the extent to which the National Health Service (NHS) in the UK is embracing this new health technology in practice. This study aimed to investigate Scottish health service infrastructure and policies that promote or impede the implementation of cCBT in the NHS.

**Methods:**

A telephone survey of lead IT staff at all health board areas across Scotland to systematically enquire about the ability of local IT infrastructure and IT policies to support delivery of cCBT.

**Results:**

Overall, most of the health boards possess the required software to use cCBT programmes. However, the majority of NHS health boards reported that they lack dedicated computers for patient use, hence access to cCBT at NHS sites is limited. Additionally, local policy in the majority of boards prevent staff from routinely contacting patients via email, skype or instant messenger, making the delivery of short, efficient support sessions difficult.

**Conclusions:**

Conclusions: Overall most of the infrastructure is in place but is not utilised in ways that allow effective delivery. For cCBT to be successfully delivered within a guided support model, as recommended by national guidelines, dedicated patient computers should be provided to allow access to online interventions. Additionally, policy should allow staff to support patients in convenient ways such as via email or live chat. These measures would increase the likelihood of achieving Scottish health service targets to reduce waiting time for psychological therapies to 18 weeks.

## Background

The National Institute of Clinical Excellence (NICE) and Scottish Intercollegiate Guidelines Network (SIGN) recommend guided self-help (GSH) and computerised cognitive behavioural therapy (cCBT) for the treatment of depression [1], anxiety [2] and bulimia nervosa [3]. In Scotland, the SIGN guidelines are especially influential and again recommend routine use of cCBT for depression [4].

The main benefit of cCBT is that it reliably delivers cognitive behavioural therapy (CBT) content in a consistent way that adheres to the CBT model, and is suited to use as part of a stepped care model. Stepped care is a flexible model of healthcare delivery in which patients can begin their treatment with a low intensity intervention requiring only limited practitioner support such as guided self-help. These are offered to those with non complex presentations and patients only ‘step up’ to longer (high intensity) specialist face to face therapy if improvements are not seen after a given time [5]. Delivering cCBT as part of a stepped care approach increases service capacity and provides an evidence-based intervention for those individuals with mild to moderately severe depression ,for example, who are otherwise least likely to receive access to specialist CBT [6].

Patients can work through the computerised packages (online or in CD-ROM format) independently and receive minimal input from the clinician. Short support sessions are key to maximising outcomes, especially for those with depression [7] and can be provided by a range of healthcare professionals and volunteers via telephone, email, text, instant messenger and Skype; or in brief face to face meetings. cCBT is a valuable alternative to self-help books (bibliotherapy) as it provides another way of accessing help that brings together multiple ways of learning - audio and video clips plus a level of interactivity. Overall cCBT has similar clinical effects as bibliotherapy [7]. User choice is one of the key factors affecting use of cCBT, a treatment option that may be particularly appealing to young people [8]. An argument for offering online cCBT is that users can access online resources at a time and place (e.g. home) they find convenient, thus fitting with busy or complex lives.

Scotland is one of the member nations of the United Kingdom with a population of 5.2 million [9]. There are many rural settlements in Scotland, however around 70% of the population live in the central lowlands in cities such as Glasgow and Edinburgh and surrounding towns. Scotland has a devolved government with control over some key policy areas including health care. NHS health boards are responsible for health policy implementation based on the needs of their respective populations and also provide management and performance monitoring within the 14 regions of the NHS in Scotland. Health boards often work with local councils and voluntary organisations in order to provide the care to patients within their region. The largest health board in Scotland is NHS Greater Glasgow and Clyde which serves 1.2 million people and employs in excess of 40, 000 members of staff across locations including 36 hospitals, 25 mental health resource centres and 298 GP surgeries. In contrast, smaller health board such as Orkney and Shetland serve significantly smaller populations of around 20,000.

There has been a commitment from the Scottish government and health policy makers to improve health and communication technologies within the National Health Service (NHS) in Scotland [10]. The government also aims to increase access to psychological therapies. This includes a specific target to reduce waiting lists for psychology services to 18 weeks by 2014 [11]. The potential for cCBT to reduce waiting lists for specialist therapy is demonstrated in a study of an online CBT package carried out by Learmonth, Trosh, Rai, Sewell and Cavanagh [12] in which only 30% of 6000 patients needed face to face therapy following use of the online intervention. cCBT is one of a range of therapies offered within the NHS and there is clearly a potential for online guided self-help interventions for common mental health problems to help to reduce therapist input and alleviate the burden on services. This approach could potentially be applied nationally by providing access to cCBT within the NHS in Scotland, thus potentially decreasing the time that individuals have to wait to receive access to an evidence based psychological treatment.

A number of issues would need to be addressed in order to provide such access to online treatment. An argument for cCBT delivered online is that patients can access the resource at a time and place convenient to them, with the assumption this will often be at home. However, the recent 2009–2010 Scottish Household Survey found that at the end of 2010, 30% of Scottish households did not have home internet access. Additionally, the survey results showed that those in deprived areas are less likely to have internet access at home. The impact of deprivation and income on internet access is clearly seen in the fact that 97% of households with annual income of over £40,000 have home internet access compared to just 35% of households with annual income of £6,001-£10,000 [13]. If health boards fail to provide access to dedicated patient computers in the various NHS buildings then these poorer individuals may miss the opportunity to engage with cCBT approaches if they were only offered via online access. This highlights the impact of digital exclusion on health and wellbeing. Other options such as using internet cafes or public libraries may not be attractive for reasons of access, privacy and stigma. In delivering cCBT it is therefore important for services to offer the option of access via existing National Health Service (NHS) facilities.

Currently there is no national policy in Scotland regarding the use of cCBT and therefore decisions are often made at a local level. Therefore access to cCBT varies across the 14 health boards in Scotland. The aim of the current study was to assess the extent to which the NHS in Scotland is providing routine access for patients to utilise cCBT. We therefore surveyed all 14 health boards in Scotland to examine access to cCBT and to better understand how health service infrastructure and information technology (IT) regulations impact on its use.

## Methods

### Design

A telephone survey investigating the technology, infrastructure and IT regulations in each of the fourteen health boards across Scotland was completed. Data collection was carried out by a qualified doctor (DK) with an interest in online health technology. IT managers or IT security managers at each of the health boards were identified and approached. A standardised proforma was used to structure the interview which generally took 5–10 minutes in total.

### Participants

The sample comprised of 14 IT managers/IT security managers from all 14 health boards across Scotland. As the sample included NHS staff members and no patients were involved in the survey, NHS research ethics approval was not needed for the study.

### Procedure

Each board area was contacted by phone between September 2010 and March 2011, and survey questions were answered by either the health board IT manager or head of IT security. Participants completed a 10 point survey, covering key areas of interest. These included routine patient access to computers within their given health board region, specification of computers and ability to meet various technical standards commonly found within cCBT packages. An open ended question was included at the end of the survey and invited participants to add any additional comments relevant to the topic of investigation and their thoughts on IT availability in the NHS.

## Results

The results of the study have been analysed and summarised under three main categories: **1. Access**; referring to whether patients are routinely able to access dedicated health service computers thereby allowing access to cCBT locally, **2. Contact**: the form of contact and support NHS staff are allowed or able to offer patients (e.g. do NHS local policies allow use of approaches such as email or Skype?) and **3. Specifications**: the technological capabilities of the computers that the health boards have (such as ability to run Flash, which is widely used to deliver video and interaction, read pdf handouts or use Java, which allows online questionnaires to be answered). Results are summarised below Table 1.

**Table 1:** Summary of findings

	**Participant responded 'yes'**	**Participant responded 'no'**
**Patient access to PCs**	5	9
**Allow access to CBT sites**	10	4
**Allow email to patients’ private account**	9	5
**NHS approved email account for patients**	1	13
**Allow Skype/MSN**	1	13
**Meet specifications for CBT sites**	14	0
**Allow flash video**	12	2
**Allow PDF**	14	0
**Allow Java**	14	0

### Access

There is a distinct lack of personal computers (PCs) dedicated for patients use within the majority of health board services in Scotland. Only 5 (36%) of the 14 health board regions in Scotland provide routine PC access to patients. Even in those providing PCs for patient use, this was often limited with one board area having dedicated computers in only one of their hospitals. At that location, use of the computer was closely observed by staff which may be off-putting to users addressing essentially private and sometimes stigmatising issues.

The nine remaining board areas did not have computers dedicated for patients use and therefore there are no definite facilities for using cCBT within NHS buildings in 64% of health board regions - covering the majority of the Scottish population.

The next question addressed whether, in theory, patients would be allowed to access CBT websites in the NHS buildings in each health board. This question was asked irrespective of whether the board actually provided computers and aimed to establish whether local information technology (IT) policy would allow patient access to cCBT. 10 (71%) boards said they would allow access to CBT sites from their computers, 4 (29%) said they would not.

### Contact with patients

The second area investigated was the ways in which practitioners and healthcare workers can provide active support and guidance for cCBT as recommended by NICE. Most health boards (n = 13 –93%) did not allow contact with patients via Skype or MSN messenger. Additionally, over a third of health boards (n = 5) do not allow practitioners to contact patients’ personal email accounts. This restriction is compounded by the fact that only 1 of the 14 (7%) of health board regions said that they can provide patients with NHS approved email accounts for correspondence with healthcare professionals.

### Specification of NHS computers

All health board regions in Scotland stated that they have computers that meet the specification required for the use of cCBT websites. All reported that their computers allow PDFs to be opened and Java to be used. Additionally, the majority (n = 12) have computers that allow flash video (which commonly delivers audio and video) in principle, however only for approved sites via web-filtering. Therefore specific websites could be made available if approved. These elements are essential aspects of many cCBT products.

### Comments from IT managers

IT managers / IT security specialists were asked for any additional or subjective comments after completing the survey. IT departments seem to be well versed in issues of patient access to IT technologies, with several responders suggesting that they find current practice restrictive and would welcome further guidance on, or revision of current regulations, particularly those governing staff-patient contacts via IT technologies. Poor bandwidth was also widely recognised as a limiting factor in streaming audio and video, which often results in health board-wide restrictions on data streaming over the internet to preserve bandwidth for routine clinical activities. One suggested solution to this issue was that cCBT data files (ie. video / audio files) could potentially be loaded directly on to dedicated patient PC clusters and streamed locally, thus avoiding issues of limited network bandwidth.

## Discussion

Guided/supported cCBT is recommended in the NICE and SIGN guidelines for the online treatment of a number of mental health problems. This approach can help improve outcomes, save service resources, increase independence and control for the patients and provide greater choice in the type of treatment patients receive. cCBT has the potential to reduce waiting times for access to evidence based psychological therapy and is delivered in ways that engage and make access easy for patients. This is especially important currently in Scotland where minimum waiting time targets (18 weeks) for psychological treatments have recently been published.

Our national survey shows that access to cCBT within NHS settings in Scotland is rarely routinely and easily available. This would especially affect those who are interested in this treatment but lack internet access at home. Our survey indicates that although most health boards have computers with adequate technological capabilities for the provision of cCBT with respect to the software required, 64% of health boards do not provide dedicated computers for patient use. This is important because this may adversely impact on those with the lowest incomes such as the low paid, unemployed and elderly who most lack access to computers and the internet [13].

Furthermore, findings show that practitioners are also limited in the extent to which they can support patients’ use of cCBT. This may limit practitioners’ ability to provide the guidance and support patients need to get the most from cCBT. Our survey shows that 36% of boards do not permit practitioners to email patients’ private email accounts. An alternative would be to provide patients with NHS email accounts for correspondence with practitioners. However, only one health board in Scotland provides this service, meaning that a large proportion of services in Scotland are left unable to provide email support to patients using cCBT. The support element is further limited by the fact that most practitioners are not permitted to utilise Skype or live chat in supporting patients. This means that support sessions may have to be delivered over the phone or during face to face appointments which limits the flexibility and convenience of the approach and may reduce its appeal to patients and practitioners.

It is encouraging that all of the NHS health boards in Scotland have PCs that meet most of the technical specifications required to deliver cCBT. We did not enquire specifically about average bandwidth available, however comments were made that this varies enormously across sites. Anecdotal evidence is that website loading times can be so poor in many routine NHS sites that viewing simple single web pages with no audio or video can take some minutes to load. The suggestion that cCBT modules could be loaded onto computers and used offline is a possibility, however this raises the issue of whether cCBT manufacturers would support their software being delivered using this method.

The present survey focussed on online delivery of cCBT materials and associated support options. Future studies could ask about other modes of supporting cCBT delivery such as telephone, text messages, and face to face contact. A further limitation of the study is that although participants clearly had significant expertise and knowledge of overall IT policy and infrastructure within their region, the complex nature of any major organisation may preclude perfect knowledge of every use of IT systems within it. The aim of our research was to establish the current routine implementation of IT within health boards, which participants were clearly well versed in.

Some health boards currently have the key components of successful cCBT delivery in place. They allow patient access to computers capable of running cCBT packages and also convenient interaction between patients and staff to maximise outcomes. However, this survey has identified a need to increase access to patient computers within NHS sites for those patients who do not have home internet access and therefore reduce the exclusion of those from deprived areas with no home internet access.

## Conclusions

This nationwide survey used the expertise of IT staff to provide vital information concerning the potential implementation of cCBT in the NHS. Although we were unable to externally verify the position of the IT employees surveyed, it was clear that all responders had the required knowledge of IT policy to contribute to the survey and share their knowledge of NHS IT policy. The results showed that despite the commitments to and potential benefits of new technology in the NHS across the UK, it is clear ‘that its adoption within the health care sector is slow and disparate’ [14]. The current survey supports this view and it is clear that for the computerised self-help approach to be truly successful, practical measures need to be taken to increase access to patient computers, and additionally practitioners need the freedom to communicate with and support their patients through a wider variety of mediums.

It is therefore not just an issue of investing in new technology but also increasing access to the equipment, resources and staff already available. Some health board regions have made these changes but many have not. This involves changes of policy to increase flexibility for staff and patients alike in accessing and supporting cCBT resources. Bandwidth issues may also need to be addressed for some locations. Future studies should continue to investigate accessibility to cCBT within health boards and review whether the delivery of cCBT has moved forward.

## Competing interests

CW is an author of a variety of written and cCBT resources. These are licenced through Five Areas Ltd, a company that delivers free and licensed online life skills resources based on a CBT model in a variety of settings including the NHS in Scotland. CW was involved in the design of the study, but he was not involved in the data collection or analysis. CAM is registered for a PhD with the University of Glasgow and was employed by both the University of Glasgow and Five Areas Ltd during the course of this research. The authors declare that they have no competing interest. CW and CAM have declared competing interests. DW declares that he has no competing interests.

## Authors’ contributions

DK designed the study, collected and analysed the data and contributed to the writing of the paper. CW was the primary investigator of the project and made a substantial contribution to the study design and writing of the paper. CM was responsible for the interpretation of the data and had a key role in writing the paper. All authors read and approved the final manuscript.

## Pre-publication history

The pre-publication history for this paper can be accessed here:

http://www.biomedcentral.com/1472-6947/12/102/prepub
